# A Systematic Sensitivity Study on Surface Pixel Shifts in High Spatial Resolution Satellite Images Resulting from Atmospheric Refraction in the Sensor to Surface Ray Path

**DOI:** 10.3390/s20236874

**Published:** 2020-12-01

**Authors:** Bo-Cai Gao, Evan Ward, Jeffrey Bowles, Adam Yingling

**Affiliations:** 1 Remote Sensing Division, Code 7230, Naval Research Laboratory, Washington, DC 20375, USA; jeffrey.bowles@nrl.navy.mil; 2 Space Sciences Division, Code 8000, Naval Research Laboratory, Washington, DC 20375, USA; Evan.Ward@nrl.navy.mil (E.W.); adam.yingling@nrl.navy.mil (A.Y.)

**Keywords:** remote sensing, sensors, atmospheric refraction, LOWTRAN

## Abstract

When viewing Earth’s surfaces from a low Earth orbiting (LEO) satellite platform with an optical sensor, the upward light propagation path from the ground to the satellite is affected by atmospheric refraction. For imaging sensors with a spatial resolution of about one km on the ground, atmospheric refraction is typically neglected during geo-registration of the satellite images. However, for high spatial resolution imaging systems with surface pixel sizes of approximately one meter or finer, the neglect of atmospheric refraction effects can typically introduce errors of a few meters in the spatially registered images. The atmospheric refraction effects need to be properly taken into consideration during the spatial registration of high spatial resolution satellite images. We have found that, with minor modifications, the ray tracing models implemented inside the LOWTRAN series of atmospheric radiative transfer codes developed in the 1970s and 1980s, in particular LOWTRAN7 in late 1980s, can be used for modeling the pixel displacement resulting from atmospheric refraction for satellite observations. The LOWTRAN series models were originally designed for calculating atmospheric transmittances and radiances for radiation going through long paths of the Earth’s atmosphere. In the ray tracing portions of the codes, a spherical model atmosphere from the ground to 100 km is finely divided into about 30 thin atmospheric layers. The refraction angles for ray paths between consecutive layer boundaries are accurately calculated. We make a new use of the refraction angles calculated by the LOWTRAN7 code to study the surface pixel shift resulting from atmospheric refraction for satellite observations. In this letter, we report the modeling results on surface pixel displacements for different satellite altitudes and downward view zenith angles, several atmospheric temperature and pressure profiles, a few surface elevations, and wavelength dependencies from blue (450 nm) to near-IR (865 nm). These results can have reference values for researchers to estimate refraction-induced pixel displacements in their high spatial resolution satellite images. The results may also potentially help in designing spacecraft algorithms for accurate instrument pointing and mission tasking to automatically capture short-lived science events.

## 1. Introduction

At present, remote Earth observations on the global scale have been made operational with the NASA Moderate Resolution Imaging Spectroradiometer (MODIS) [1,2] and Visible Infrared Imaging Spectroradiometer Suite (VIIRS) [3] instruments from polar orbiting satellite platforms. The upward ray paths from surfaces to satellites are affected by atmospheric refraction effects. In order to find out if the refraction effect should be taken into consideration for the precise geolocation of MODIS and VIIRS imaging data, Noerdlinger [4] developed an analytic method that determined the angle of atmospheric refraction and the apparent displacement of the point of Earth intersection due to atmospheric refraction. In the Noerdlinger method, a single layer and a spherically symmetric model atmosphere are assumed. The conversion of the bending angle to horizontal pixel displacement is made approximately by assuming a scale height for the troposphere. The results from Noerdlinger’s simulations show that, for a zenith angle of approximately 45 degrees and a mean tropospheric scale height of 10.5 km, a pixel in a satellite image at sea level would be displaced by about 5 m, which is much smaller than the pixel sizes of about one km for some MODIS bands and 750 m for some VIIRS bands. As a result, in the present MODIS and VIIRS geolocation data products, the atmospheric refraction effects are neglected.

In recent years, high spatial resolution satellite images with spatial resolutions of ~1 m or finer are increasingly available for public use. The accurate geolocation of high spatial resolution images requires explicit consideration of atmospheric effects. A review of atmospheric refractive electromagnetic wave bending and propagation delay was given by Mangum and Wallace [5]. They suggested that, for accurate calculation of the refractive bending, a two-layer model atmosphere (a stratosphere layer on top of the troposphere layer) is required. Yan et al. [6] reported a method for the correction of the geolocation error resulting from atmospheric refraction for high resolution optical satellite pushbroom images. They used a simplified two-layer model atmosphere, including troposphere plus stratosphere, to calculate the refractive bending effects. Their calculated total surface pixel displacement resulted from the combination of tropospheric bending and stratospheric bending. The work cited above on modeling the atmospheric refractive bending effects comes from the astronomy and satellite geodesy research communities.

Since the early 1970s, a number of ray tracing algorithms assuming multi-layered spherically symmetric model atmospheres (~30 layers) with refraction have been under development in the atmospheric sciences community [7,8,9]. The algorithms are used mainly for the accurate calculation of solar ray path length in each layer of the atmosphere for light reaching the ground, high altitude balloons [10,11], or satellite [12] instruments under low sun angle conditions. The calculated path lengths in the multi-layered model atmosphere are then used for converting the retrieved amounts of trace atmospheric gases in the long slant paths from measured infrared transmission spectra to equivalent amounts of gases in vertical paths from the ground to space [12].

The development of the “Airmass Computer Program for Atmospheric Transmittance/Radiance Calculations: FSCATM” in 1983 [9] marked the maturity of ray tracing algorithm development in the atmospheric sciences community. The FSCATM ray tracing algorithm with minor improvements was incorporated into a number of atmospheric modeling algorithms, such as LOWTRAN6 [13], LOWTRAN7 [14], and MODTRAN [15]. It allows several options for specifying the slant path geometry, including the path between a satellite altitude (H_1_) and a ground surface altitude (H_2_). Based on our careful study of the publicly available LOWTRAN7 computer source code, we have found that the refraction angles for ray paths between consecutive atmospheric layer boundaries are accurately calculated, but these angles are not written out in the intermediate printing files with enough useful digits. We make a new use of the LOWTRAN7 code to calculate refraction angles for the study of surface pixel shift resulting from atmospheric refraction for satellite observations. In this letter, we report the modeling results on surface pixel displacements for different satellite altitudes and downward view zenith angles, several atmospheric temperature and pressure profiles, a few surface elevations, and wavelength dependencies from blue (450 nm) to near-IR (865 nm).

## 2. Methods

The multi-layered atmospheric ray tracing algorithm used in this study has been well described previously in several scientific reports [9,13,14]. Below, we summarize the main points on the ray tracing algorithm implemented inside LOWTRAN7.

Within LOWTRAN7, there are a total of 6 built-in model atmospheres that correspond to the US Standard (1976), tropical, mid-latitude summer, mid-latitude winter, sub-arctic summer, and sub-arctic winter models. For each model atmosphere, the altitude, pressure, temperature, and water vapor density vertical profiles are tabulated in 1 km steps from 0 to 25 km, 5 km steps from 25 to 50 km, and then at 70 km and 100 km. The last altitude level is specified at 99,999 km, which accommodates the above atmosphere satellite viewing geometry. LOWTRAN7 also permits users to input their own model atmospheres.

**Figure 1:**
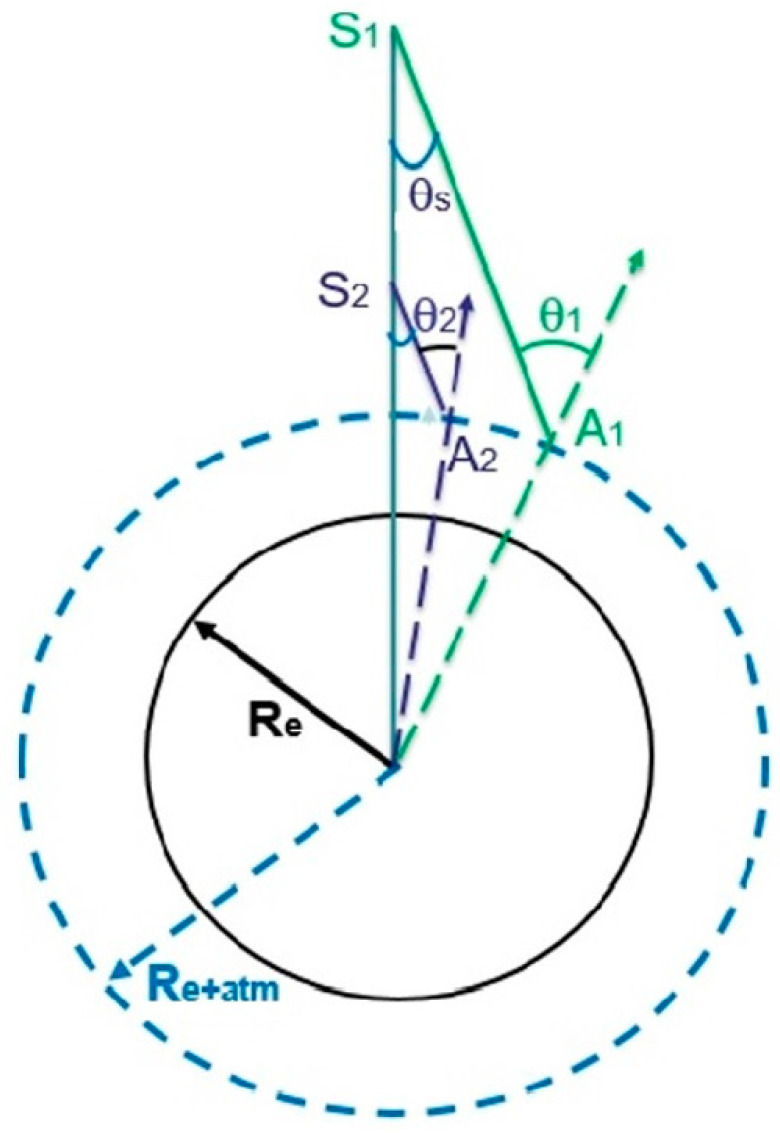
A diagram illustrating the satellite-surface viewing geometry. It is noted that, for satellites at different altitudes with the same downward view zenith angle, the view zenith angles at the top of the atmosphere are different.

Figure 1 is a diagram illustrating the satellite-surface downward viewing geometry. The solid black circle represents the spherical Earth with a radius of *R_e_*. The dashed circle represents the top of the Earth’s atmosphere (TOA) with a radius of *R_e_* plus the 100 km thickness of a model atmosphere. Suppose that two satellite sensors located at *S*_1_ and *S*_2_ with *S*_1_ > *S*_2_, but with the same satellite downward viewing zenith angle *θ_s_*, are looking at the Earth’s surface. The intersecting points at TOA are *A*_1_ and *A*_2_, respectively. The local upward view zenith angles at *A*_1_ and *A*_2_ are *θ*_1_ and *θ*_2_, respectively, but with *θ*_1_ > *θ*_2_ due to the Earth’s curvature. The ray path from *S*_1_ to the Earth’s surface will have larger refractive displacement than the path from *S*_2_ to the surface because of a greater local incident angle at *A*_1_. Refractive ray path geometries have also been illustrated in many other papers and reports, such as those of Noerdlinger [4], Yan et al. [6], and Kneizys et al. [13]. Those illustrations generally do not reflect explicitly the fine point that the amount of surface pixel displacement resulting from atmospheric refraction is dependent on the satellite altitude.

After a ray path reaches the top of the model atmosphere at 100 km, the refracted ray path within the atmosphere is traced layer by layer, according to Snell’s law [9,16].

*n_i_* (*R_e_* + *Z_i_*) *sin*(*θ_i_*) = constant (1)

where *n_i_* is the air refractive index for the *ith* level of a model atmosphere, *R_e_* is the Earth’s radius, *Z_i_* is the level height above the ground, and *θ_i_* is the local zenith angle for the *ith* level [14].

The air refractive index from Edlen [17] is used in LOWTRAN7. The formulation is given by:

(n − 1) × 10^6^ = {a_0_ + a_1_/[1 − (ν/b_1_)^2^] + a_2_/[1 − (ν/b_2_)^2^]} × (P − P_w_)/P_0_ × 296.15/T + [c_0_ − (ν/c_1_)^2^] × P_w/_P_0_ (2)

where ν is the wavenumber in cm^−1^, P is the total pressure in mb, P_w_ is the partial pressure of water vapor, P_0_ is equal to 1013.25 mb, T is the temperature in K, and the constants a, b, and c are as follows:

a_0_ = 83.43, a_1_ = 185.08, a_2_ = 4.11 b_1_ = 1.14 × 10^5^, b_2_ = 6.24 × 10^4^ c_0_ = 43.49, c_1_ = 1.7 × 10^4^ (3)

LOWTRAN7 assumes a spherically symmetric atmosphere with exponential profiles of density and a refractive index between layer boundaries. Such an implementation scheme allows accurate refractive ray path calculations for different viewing geometries [9,14].

Within LOWTRAN7, there are three types of built-in viewing geometries. Type 2 permits the specification of a slant or a vertical path between two altitudes. We have made use of this option by specifying the path between a satellite altitude (*H*_1_) with a satellite downward viewing zenith angle (*θ_s_*) and a ground surface altitude (*H*_2_). Through careful studies of the publicly available LOWTRAN7 computer source code and the sample input and output files, we have found that the refractive bending angles for ray paths between consecutive layer boundaries are accurately calculated, but these angles are not written out in the intermediate printing files with enough useful digits. We have made minor modifications to the LOWTRAN7 source code so that the refractive bending angles are properly outputted. Using the layer by layer bending angles, view zenith angles, and layer heights above the ground, the surface pixel displacements for all atmospheric layers are calculated with the formulation used by Yan et al. [6]. The summation of the displacements resulting from the 32 layers of the model atmosphere yields the total refractive surface displacement from the satellite to the ground.

## 3. Results

We have made extensive calculations of refractive surface pixel displacements using the modified version of the LOWTRAN7 code for blue (450 nm), green (550 nm), red (650 nm), and near IR (NIR) (860 nm) channels with a narrow width of 10 nm, satellite altitudes of 300, 400, 500, 600, 700, and 800 km, satellite downward viewing zenith angles between 5 and 80 degrees, three model atmospheres (the 1976 US Standard, tropical, and sub-arctic winter models), and five surface elevations at 0, 1, 2, 3, and 4 km. Sample calculation results are presented below.

### 3.1. Atmosphere Height Versus Layered Green Light Bending Angle and Cumulative Shift at Surface

**Figure 2:**
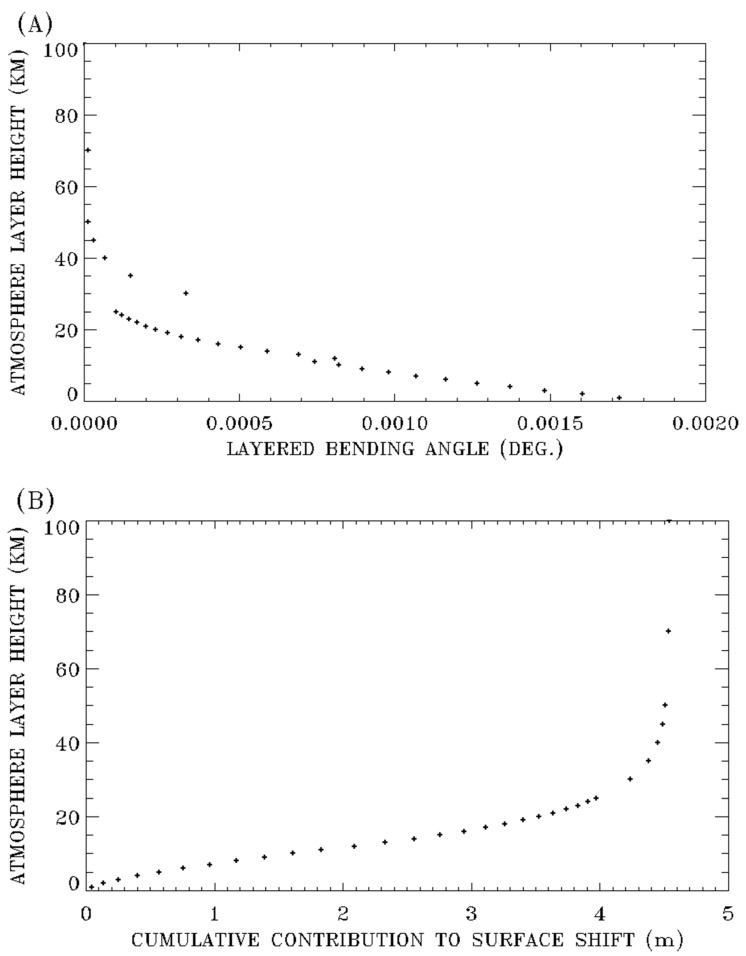
(**A**) Layer by layer atmospheric refractive bending angles and (**B**) cumulative pixel shift at the surface for green light (550 nm) for the US 1976 model atmosphere, a satellite altitude of 500 km, and a downward view zenith angle of 45 degrees.

Figure 2A shows a sample plot for atmosphere height as a function of the layered green light bending angle. The plot is calculated for a satellite altitude of 500 km, a satellite downward view zenith angle of 45 degree, the 1976 US Standard model atmosphere, and a surface elevation of 0 km. At the lower altitudes below 5 km, the layered bending angles are relatively large in comparison with those in the 10–15 km altitude range. The bending angle at 30 km suddenly jumps up because the model atmosphere thickness is increased to 5 km, instead of the 1 km thickness for layers below 25 km.

Figure 2B shows the corresponding plot for atmosphere height as a function of cumulative contribution to surface shift. The total surface shift in this case for the satellite to surface ray path is calculated to be 4.54 m. The atmosphere below 30 km contributes approximately 93% to the total surface shift. By comparing Figure 2B with Figure 2A, it is seen that, although the layered bending angles in the 0–5 km altitude range are greater than those in the 10–15 km range, the contributions to surface shift from the 0–5 km altitude range are smaller than those from the 10–15 km altitude range. The reason is that if two layers have the same bending angle, the higher the layer altitude is, the greater the contribution to surface shift is.

For validation purposes, we compared the calculated total shift with the shift reported by Noerdlinger [4] for very similar observational geometries. Our reported total shift for the satellite altitude of 700 km and the downward view zenith angle of 45 degrees is 5.13 m (for all 32 finely divided atmospheric layers from the ground to 100 km). The shift reported by Noerdlinger [4], with the assumptions that the calculation was actually made for the MODIS altitude of 705 km and for the one-layer model atmosphere, is 5.46 m. For the satellite downward view zenith angle of 50 degrees, the shifts from the LOWTRAN7 simulation and from Noerdlinger [4] are 7.70 m and 7.73 m, respectively. For the satellite downward view zenith angle of 55 degrees (the edge of a MODIS scan line), the shift from LOWTRAN7 and that from Noerdlinger [4] are 13.14 m and 11.40 m, respectively. Overall, the LOWTRAN7 simulation results agree quite well with those from Noerdlinger [4].

### 3.2. Dependence of Refractive Surface Shifts on Sensor View Zenith Angles and Satellite Altitudes

**Figure 3:**
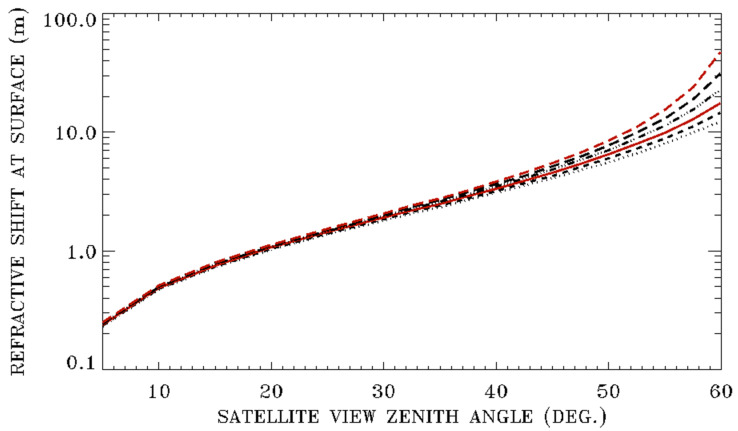
From the top to bottom curves—refractive surface pixel shifts versus satellite downward view zenith angles for satellite altitudes of 800, 700, 600, 500, 400, and 300 km, respectively, for green light (550 nm) and the US 1976 model atmosphere.

Figure 3 shows six curves of refractive surface pixel shifts versus satellite downward view zenith angles for satellite altitudes of 800, 700, 600, 500, 400, and 300 km. The calculations are made for green light at 550 nm for the 1976 US Standard model atmosphere and a surface elevation of 0 km. From these curves, it is seen that the refractive surface pixel shifts are approximately 1 m for a satellite downward viewing zenith angle of 20 degrees, regardless of the satellite altitude. However, for satellite downward view zenith angles greater than about 40 degrees, the higher the satellite altitude is, the greater the refractive surface pixel shift is. Below are two specific examples. For a satellite at 300 km with a downward viewing zenith angle of 60 degrees, the refractive shift at the surface is 12.53 m. On the other hand, for a satellite at 800 km with the same view zenith angle, the refractive shift is increased to 48.83 m. The increase of the refractive shift at the surface with the growth of the satellite altitude and satellite view zenith angle is mainly due to the Earth’s curvature effect. At a higher satellite altitude and a larger satellite downward view zenith angle, the ray path between the satellite and the top of the atmosphere (TOA) (100 km) has a larger local view zenith angle at TOA (see Figure 1), and the subsequent ray path from TOA to the Earth’s surface is subjected to more atmospheric refractive bending. The curves in Figure 3 may have reference values for satellite algorithm designers to decide if the atmospheric refractive effects should be taken into consideration when designing spacecraft algorithms for accurate pointing and mission tasking of available high spatial resolution images with surface pixel sizes in the order of 1 m or finer so that short-lived small scale science events can be automatically captured.

### 3.3. Dependence of Refractive Shifts on Model Atmospheres

**Figure 4:**
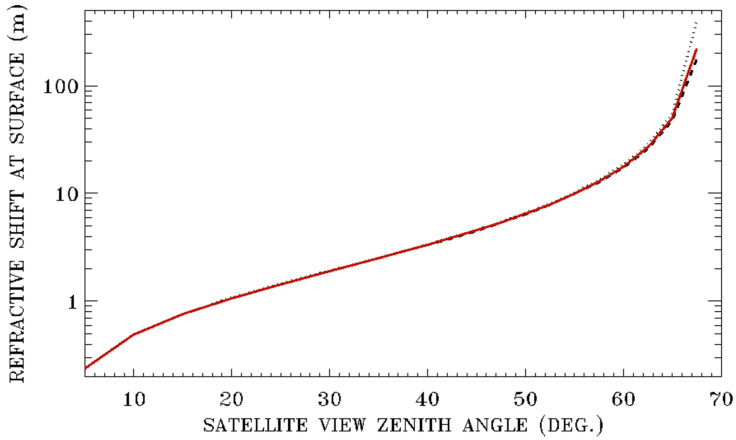
From the top to bottom curves—refractive surface pixel shifts as a function of satellite downward viewing angles for the sub-arctic winter, US 1976 standard, and tropical models, respectively. The calculations are made for green light at 550 nm and a satellite altitude of 500 km.

According to Equation (2), the air refractive indices depend on temperature, pressure, and partial pressure of water vapor in the atmosphere. In Figure 4, we show surface pixel shifts as a function of satellite downward viewing angle for three model atmospheres, i.e., the sub-arctic winter, US 1976 standard, and tropical models. The sub-arctic winter model atmosphere represents very dense and dry atmospheric conditions. The US 1976 standard model atmosphere represents average atmospheric conditions, while the tropical model atmosphere represents hot and moist tropical summer conditions. Additionally, the three curves in Figure 4 are calculated for green light at 550 nm, a satellite altitude of 500 km, and a surface elevation of 0 km. For satellite downward view zenith angles below 60 degrees, the three curves do not have much separation. At the satellite downward view zenith angle of 60 degrees, the surface pixel shifts for the sub-arctic winter, US 1976, and tropical model atmospheres are 18.51, 17.79, 17.48 m, respectively. This indicates that surface pixel shifts resulting from refractive bending are very weakly dependent on atmospheric models. As a result, satellite algorithm designers may not need to take detailed consideration for atmospheric conditions when designing spacecraft algorithms for instrument pointing for satellite downward view zenith angles less than about 60 degrees. At a satellite downward view zenith angle of 65 degrees, the surface pixel shifts for the same three model atmospheres are 55.68, 50.12, and 47.64 m, respectively. Therefore, in order to achieve a pointing accuracy of about 1 m, satellite algorithm designers may need to have detailed consideration for atmospheric conditions for satellite downward viewing angles greater than about 60 degrees.

### 3.4. Dependence of Refractive Shifts on Wavelengths

**Figure 5:**
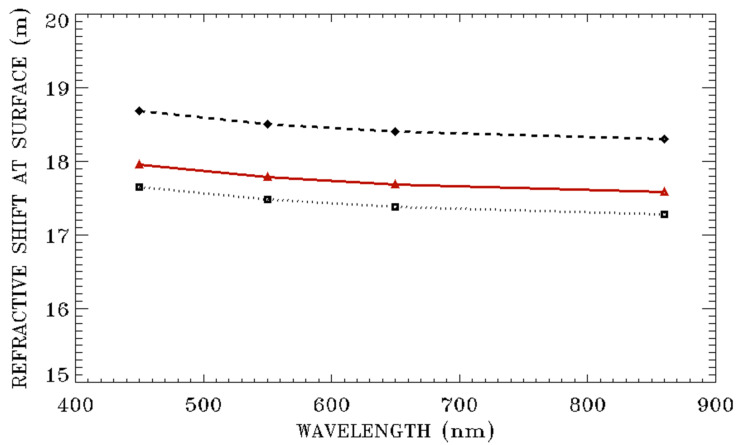
From the top to bottom curves—refractive surface pixel shifts as a function of wavelength for the sub-arctic winter, US 1976 standard, and tropical model atmospheres, respectively. The calculations are made for a satellite altitude of 500 km and a satellite downward view zenith angle of 60 degrees.

In Equation (2), wavenumber ν is equal to the inverse of wavelength (λ), i.e., ν = 1/λ. Therefore, the air refractive indices are wavelength dependent. In Figure 5, we show the surface pixel shifts as a function of wavelength for three model atmospheres. The calculations are made for a satellite altitude of 500 km and a large satellite downward view zenith angle of 60 degrees. For each of the curves in Figure 5, the dependence of surface pixel shift on wavelength is quite small. By comparing the curve for the sub-arctic winter model with that for the tropical model, it is seen that the higher surface pressure sub-arctic winter model introduces about 1 m more refractive shift for the large view zenith angle of 60 degrees and for blue, green, red, and NIR band lights.

### 3.5. Dependence of Refractive Shifts on Surface Elevations

**Figure 6:**
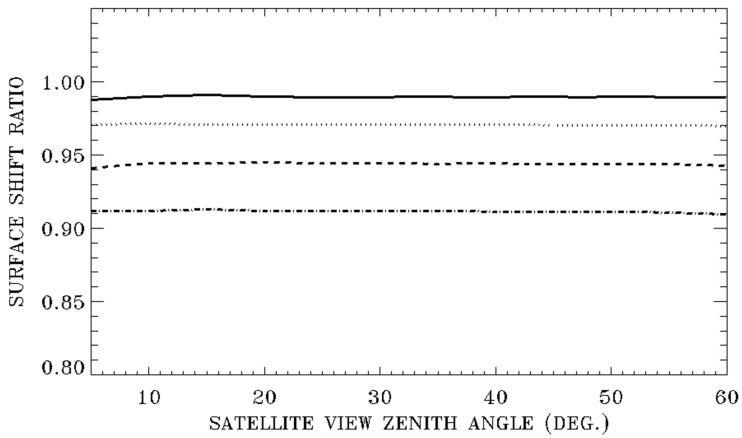
Illustrations of the dependence of refractive pixel shifts on surface elevations. From top to bottom, the curves are ratios of refractive pixel shifts for surface elevations of 1, 2, 3, and 4 km over those of 0 km, respectively.

As shown in Equation (2), the air refractive index is a function of atmospheric pressure. In general, the surface pressure decreases with increasing surface elevation. For example, over the mile-high Denver City in Colorado, the surface pressure is only about 85% of the sea level pressure. Figure 6 illustrates the dependence of refractive surface pixel shifts on surface elevations. The four curves from top to bottom in Figure 6 are ratios of pixel shifts for surface elevations of 1, 2, 3, and 4 km over those of 0 km. All curves are calculated for 550 nm green light, a satellite altitude of 500 km, the US 1976 model atmosphere, and satellite downward view zenith angles between 5 and 60 degrees. From these curves, it is seen that the refractive surface pixel shifts for surface elevations of 1, 2, 3, and 4 km decrease by approximately 1, 3, 5.7, and 9%, respectively. These decreases are nearly independent of satellite view zenith angles.

## 4. Discussions

The built-in LOWTRAN7 viewing geometries permit the direct modeling of the atmospheric bending effects for the satellite to surface ray path. However, the algorithms developed from the astronomy community [4] generally do not have satellite altitude as an explicit variable. As a result, it is not straight forward to use such algorithms to study the sensitivity of surface pixel shifts on satellite altitudes (see Figure 3), and additional coordinate rotations are needed.

## 5. Summary

Through our studies of the LOWTRAN7 computer code developed in late 1980s by the atmospheric sciences community, mainly for modeling atmospheric gaseous transmittances and radiances, we have found that the ray tracing models implemented inside LOWTRAN7 with minor modifications can be used for modeling surface pixel displacements resulting from atmospheric refraction for downward satellite to earth surface observational geometry. We have made extensive calculations of refractive surface pixel displacements using the modified LOWTRAN7 code for blue (450 nm), green (550 nm), red (650 nm), and near IR (NIR) (860 nm) channels with a narrow width of 10 nm; satellite altitudes of 300, 400, 500, 600, 700, and 800 km; satellite downward viewing zenith angles between 5 and 80 degrees; three model atmospheres (the 1976 US Standard, tropical, and sub-arctic winter models); and five surface elevations at 0, 1, 2, 3, and 4 km. The dependences of refractive surface pixel displacements on satellite altitudes, satellite downward view zenith angles, atmospheric models, wavelengths, and surface elevations are presented. These results can have reference values for researchers to estimate refraction-induced pixel displacements in their high spatial resolution satellite images. The results may also potentially help in designing spacecraft algorithms for accurate instrument pointing and mission tasking to automatically capture short-lived small-scale science events.
